# Ocular Tissue Engineering: Current and Future Directions

**DOI:** 10.3390/jfb6010077

**Published:** 2015-02-17

**Authors:** D. Karamichos

**Affiliations:** 1 Department of Cell Biology, University of Oklahoma Health Sciences Center, Oklahoma City, OK 73104, USA; E-Mail: dimitrios-karamichos@ouhsc.edu; 2 Department of Ophthalmology, Dean McGee Eye Institute, University of Oklahoma Health Sciences Center, Oklahoma City, OK 73104, USA

**Figure 1:**
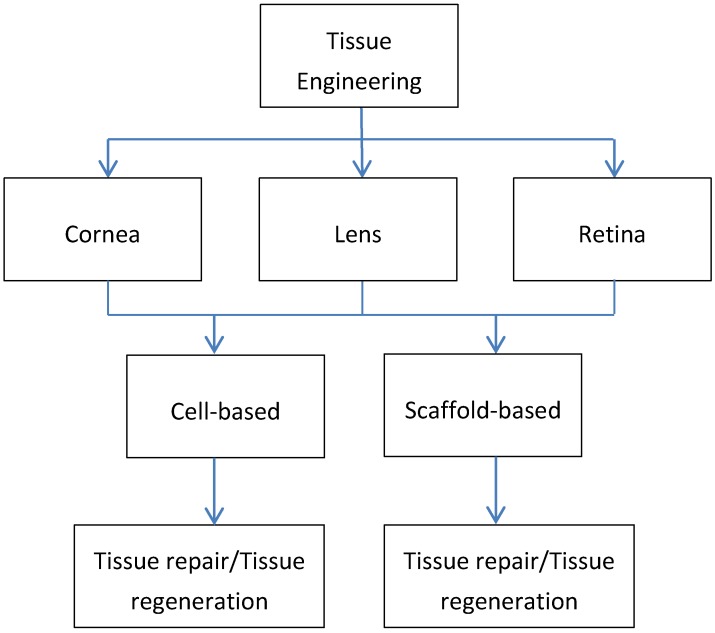
Schematic diagram highlighting the importance of tissue engineering (TE) approaches in ocular tissues: cornea, lens, and retina.

Tissue engineering (TE) is a concept that was first emerged in the early 1990s to provide solutions to severe injured tissues and/or organs [1]. The dream was to be able to restore and replace the damaged tissue with an engineered version which would ultimately help overcome problems such as donor shortages, graft rejections, and inflammatory responses following transplantation. While an incredible amount of progress has been made, suggesting that TE concept is viable, we are still not able to overcome major obstacles. In TE, there are two main strategies that researchers have adopted: (1) cell-based, where cells are been manipulated to create their own environment before transplanted to the host, and (2) scaffold-based, where an extracellular matrix is created to mimic *in vivo* structures. TE approaches for ocular tissues are available and have indeed come a long way, over the last decades; however more clinically relevant ocular tissue substitutes are needed. Figure 1 highlights the importance of TE in ocular applications and indicates the avenues available based on each tissue.

In cornea, TE approaches are vital in order to maintain the transparent barrier between the eye and the environment. Of the three corneal layers (epithelium, stroma, and endothelium) probably the most difficult one to replace is the stroma. Stroma is a thick, transparent middle layer, consisting of regularly arranged collagen fibers along with sparsely distributed resident cells commonly known as keratocytes. The corneal stroma consists of approximately 200 collagen fibril layers and account for up to 90% of the total corneal thickness. Corneal transplantation is currently the only surgical procedure for replacing damaged or diseased corneas. Damaged cornea is replaced by donated corneal tissue in its entirety (penetrating keratoplasty) or in part (lamellar keratoplasty). While the surgical procedure has been somewhat successful, major problems remain including donor corneas shortage, risks of infection, and graft rejection. In an attempt for an alternative avenue, several studies have reported successful cultivation of corneal stroma, in combination with corneal epithelium and endothelium, however the long-term *in vivo* data and clinical applications are still lacking [1]. The corneal epithelium has been targeted by scientists and a variety of TE applications using both cell and scaffold-based approaches have been developed [2,3,4,5,6]. Studies reporting the successful transplantation of mucosal epithelial cells [5,6] as well as limbal stem cells [2] are promising. Tissue grafts such amniotic membranes [3,4] have also been reported and used in humans. While these have been assessed in clinical setting, long-term studies are still needed in order to safely assess the benefits.

In lens, despite the limited number of studies developing TE solutions, there is a clear need for cataract surgeries alternatives. Currently, lens opacification or else known as cataracts are treated surgically by removing the lens and replacing it with artificial intraocular lenses (IOL) [1,7]. Most of the people receiving cataract surgery will need to come back for a second surgery due to the posterior capsule opacification (PCO). PCO occurs because lens epithelial cells remaining after cataract surgery have grown on the capsule causing it to become hazy and opaque [1,7,8]. Development of alternatives is almost nonexistent and urgently needed. One of the few TE approaches was reported by Tsionis *et al.* [9] where a human retinal PE cell line cultured in Matrigel was differentiated in lentoids and lens-like structures. Nevertheless, therapies based on this technique or others are far away and it remains unknown if TE is the future for lens related clinical problems. 

In retina, both cell and substrate-based TE approaches have been reported mainly in animal models. Homologous retinal pigment epithelium (RPE) cells have been transplanted in the subretinal space with no visual benefits to the patients [10,11]. On the other hand autologous RPE transplantation resulted in clinically significant improvement of vision; however the limited number of healthy cells that can be isolated from the patient is a huge problem [12,13]. The concept of the use of polymers for retinal TE is rather new and has only been emerged in the last decade or so. As reviewed by Trese and co-authors [14] the ideal polymer for retinal transplantation should be thinner than 50 μm, porous, biodegradable, and have the correct Young’s modulus. Several polymers fulfill this criteria including but not limited to poly(lactic-co-glycolic acid) (PLGA), poly(lactic acid (PLLA), poly(glucerol-sebacate) (PGS), and poly(caprolactone) (PCL) [14,15]. However, only a few studies have shown promising results using these or other polymers for TE retinal applications. The combination of PLLA-PLGA polymer reported by Thomson and co-authors [16] showed good RPE cellular viability, adhesion and proliferation for the course of the month long study. However, the main limitation of this study was the use of cell lines instead of primary cells which are known to be different in terms of their behavior. The general consensus is that embryonic stem cells (ESC) and induced pluripotent stem (iPS) cells are a better choice since they more closely resemble actual RPE. This, however, remains to be seen. Regardless of the cell source, technical challenges still remain before cell-substrate based therapies can be successful.

In conclusion, the human eye with the different structures, cell types, and tissues is an ideal candidate for TE approaches. The eye structures and the inadequate to-date therapies make this a very attractive tissue for TE. This is well understood within the scientific community and that is why significant discoveries and knowledge advancements have been made. Perhaps the one tissue with the most success is the corneal epithelium. There is no reason why the other structures cannot be regenerated or reconstructed using TE techniques. The challenge here is to be able to get the scientists, engineers, and clinicians to work together in order to tackle today’s challenges and give our patients the best possible treatment. 
